# Contagious charisma: the flow of charisma from leader to followers and the role of followers’ self-monitoring

**DOI:** 10.3389/fpsyg.2023.1239974

**Published:** 2023-11-02

**Authors:** Tal Katz-Navon, Marianna Delegach, Eden Haim

**Affiliations:** ^1^Arison School of Business, Reichman University, Herzliya, Israel; ^2^Sapir Academic College, Ashkelon, Israel

**Keywords:** charisma, charismatic leadership, cascading effect, self-monitoring, leadership

## Abstract

Charisma, the captivating attribute that endows an individual with the power to inspire and influence others, is frequently associated with possessing an attractive personality, effective communication skills, and the capacity to draw people in and lead them. The concept of the trickle-down effect in leadership theory suggests that the characteristics of a leader’s style including perceptions, emotions, attitudes, and behaviors, have the potential to be “contagious” and spread to their followers. Nevertheless, it is unclear whether and when a leader’s charisma may be transferred to followers, as charisma is predominantly a trait associated with the leader. Integrating insights from the social learning, emotional contagion, and self-concept theories, we propose that charisma can cascade downward from the leader to followers and that this effect is contingent on the individual follower’s level of self-monitoring. Measuring a sample of 127 followers and 15 leaders in a large organization at two time points, we found that throughout time the leader’s charisma indeed cascaded down to followers, i.e., followers of a charismatic leader were perceived as more charismatic throughout time. However, this effect was prominent only for low-monitoring followers. Novel insights into the flow-down effect of charisma, avenues for future research, and practical implications are discussed.

## Introduction

Charisma has been referred to as some unknown quality or miraculous ability (Banks et al., 2017), a “mysterious gift” (Shamir, 1992) awarded to special individuals. However, recent advances in charisma research have moved away from this trait-like mystic aura construct and have focused on the individual’s behaviors, attitudes, and emotions that are perceived by others as charismatic. Accordingly, charisma is defined as “values-based, symbolic, emotion-laden leader signaling” (Antonakis et al., 2016, p. 304), implying that charisma could potentially be learned and cultivated (Antonakis et al., 2011).

Leaders shape their followers’ leadership styles (e.g., Bass et al., 1987). This process of leadership is termed cascading effect and it illustrates how leadership behaviors flow down through hierarchical levels and positively impact the leadership behaviors of lower-level employees (Byun et al., 2018). This cascading of leadership phenomenon has been observed primarily in certain leadership styles like servant leadership (e.g., Wang et al., 2018; Stollberger et al., 2019; Lemoine and Blum, 2021), ethical leadership (e.g., Mayer et al., 2009; Schaubroeck et al., 2012; Hansen et al., 2013), and authentic leadership (e.g., Hirst et al., 2016; Zheng et al., 2022). These leadership styles are follower-centric and thus are fundamentally aimed to enhance followers’ leadership attributes. In contrast, charismatic leadership is leader-centric (Judge et al., 2002) and focuses almost solely on the leader as a central actor who affects organizational outcomes (Conger and Kanungo, 1987). Thus, although charismatic leaders’ ‘signaling can directly and indirectly influence followers’ attitudes, emotions, and behaviors (DeGroot et al., 2000), it is unclear whether the leader’s charisma can flow down to followers. Specifically, the influence of charismatic leaders, which typically results in followers embracing the leader’s vision, adhering to their guidance, and being motivated by their charisma to attain organizational goals, has been extensively documented (DeGroot et al., 2000; Banks et al., 2017). Nevertheless, the degree of transformation experienced by followers through their interactions with charismatic leaders, encompassing more profound and deeper shifts as they increasingly align with the leader’s charisma, warrants further investigation. Hence, the present study advances and empirically tests the hypothesis that a leader’s charisma can indeed be transmitted to followers. To do so, it leverages the theories of social learning (Bandura, 1977), emotional contagion (Barsade, 2002), and self-concept (Shamir et al., 1993) as a comprehensive framework.

Furthermore, the followers’ individual characteristics determine their proneness to be affected by the influence of the charismatic leader (Shamir et al., 1993; Wegge et al., 2022). Specifically, self-monitoring, which captures interpersonal variation in the degree to which individual behavior reflects social cues as opposed to inner state (e.g., Tasselli et al., 2015), has emerged as an important and relevant individual characteristic in leadership research (e.g., Day et al., 2002). High self-monitors craft their self-presentations to fit the requirements of the situation (Snyder, 1979), while those lower on self-monitoring reflect their authentic and true selves (Kudret et al., 2019). Thus, we suggest that the potential flow of charisma from leader to followers may differ between high vs. low self-monitoring individuals: low self-monitors, who rely on their inner self, are more likely to align it with the leader’s charismatic cues and thus are prone to change their charismatic behaviors, emotions, and self-concept in response to the charismatic leader’s appeal. On the other hand, high self-monitors adjust their self-presentation according to the charismatic leader’s cues, and hence their charismatic behaviors are perceived as less genuine. This authenticity is an essential attribute of charisma (Kleinbaum et al., 2015).

**FIGURE 1:**
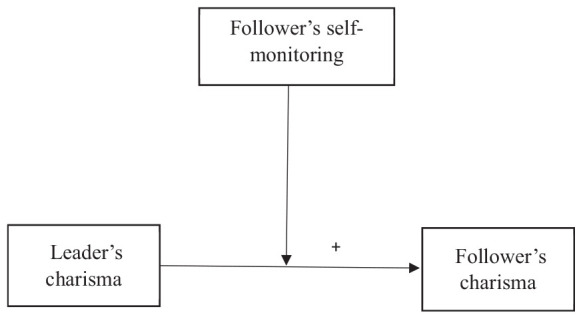
The research model.

In summary, the aims and related contributions of the current study are 2-fold. First, we expand the literature on both the trickle-down effect of leadership and on charisma by proposing a theoretical framework for the transfer of the latter from leader to followers. By examining the potential learnability and transferability of charisma (Antonakis et al., 2011), the study offers a fresh perspective on the extent to which charismatic attributes can extend from leaders to followers. It accomplishes this by introducing a theoretical framework that explores the flow of charisma across leadership tiers, suggesting a new perspective on how leadership qualities can extend beyond the leader’s role. In addition, follower characteristics are important boundary conditions for leadership (Liden and Antonakis, 2009), and elucidating boundary conditions is an essential step in theory development (Whetten, 1989). The literature emphasizes how the leader’s personality traits affect the leadership process (e.g., Judge et al., 2002). However, there is a scarcity of research on how follower personality traits may moderate a leader’s effect (Matthews et al., 2021). In this respect, our study could spark revelations about the intricate interplay between leader charisma and follower traits, contributing to the evolving landscape of charisma research. The research model outlined for this study is depicted in Figure 1.

## Literature review and hypotheses

### The flow of charisma

The trickle-down effect is defined as a flow of attitudes, perceptions, emotions, and behaviors from higher to lower hierarchical levels within the organization (Wo et al., 2019). Specifically, the trickle-down effect of leadership posits that perceptions, feelings, attitudes, or behaviors that characterize a manager’s leadership style are transferred to that of a lower-level manager, which in turn influences the followers’ feelings, attitudes, or behaviors (Wo et al., 2019)^1^. Scholars have used social learning theory (Bandura, 1977) as a framework to explain this trickle-down effect of leadership (e.g., Mayer et al., 2009; Mawritz et al., 2012; Wang et al., 2018; Xie et al., 2019). Specifically, leaders are significant social contacts. By observing and emulating the leader’s behaviors, attitudes, rhetoric, and emotional expressions, followers learn and imitate leadership-relevant norms and actions, and thus their leadership style converges around the leader’s leadership style (Bandura, 1977; Schaubroeck et al., 2012). However, the extant research that demonstrates the trickle-down effect focuses mainly on follower-centric leadership styles, such as authentic, servant, and ethical leadership (e.g., Mayer et al., 2009; Schaubroeck et al., 2012; Hansen et al., 2013; Hirst et al., 2016; Wang et al., 2018; Stollberger et al., 2019; Lemoine and Blum, 2021; Zheng et al., 2022). These follower-centric forms of leadership are essentially directed toward the followers’ growth and development; they emphasize the cultivation of positive leader-follower relationships and the coaching of followers to demonstrate prosocial behaviors, high morality, and integrity (Lemoine et al., 2019). In contrast, charismatic leadership is a leader-centric leadership style that emphasizes the leader’s qualities—traits, abilities, behaviors, and affect—making up the charisma that enables the leader to influence the followers’ outcomes. Thus, while the former specifically focuses on the leader helping and facilitating the followers’ leadership (Manz and Sims, 1991), the latter concentrates on the leader’s extraordinary qualities that enable them to inspire and emotionally arouse their followers to achieve the group’s goals (Mhatre and Riggio, 2014). Based on this fundamental difference between follower-centric leadership styles that specifically aim to develop followers’ leadership, and leader-centric leadership style that focuses on the leaders’ abilities and behaviors to affect outcomes, the question is whether charisma also “trickles down” from leader to followers since it is not the primary intention of the leader to develop followers’ charisma.

In order to answer this question and based on the three aspects of charisma - behavioral-cognitive (e.g., Antonakis et al., 2016), emotional (e.g., Bono and Ilies, 2006), and self–concept (e.g., Shamir et al., 1993), we suggest three theoretical explanations for the potential flow or transfer of charisma. First, social learning theory emphasizes that “most of the intricate responses people display are learned, either deliberately or inadvertently, through the influence of example” (Bandura, 1973, p. 44). As a significant social contact, the leader functions as a role model by representing that which is achievable and being inspirational, and by portraying aspirations, motivation, and goals (Morgenroth et al., 2015). Personal identification with the leader is one of the key influence processes in the leader-follower relationship (Conger and Kanungo, 1987). The followers perceive the charismatic leader to possess these “extraordinary” qualities and tend to identify with and admire the leader; thus, the leader becomes a point of reference for followers that defines the attitudes and behaviors that they should develop (Bass et al., 1987). Followers mimic the leader unintentionally and subconsciously (Myers, 2018), adopt the leader’s values, morals, and ideals, and enact the leader’s behaviors. This vicarious learning among followers refers to the process of absorbing and interpreting the leader’s behaviors and experiences that expands the follower’s repertoire of charismatic responses and represents the charisma “spillover” or flow from the leader to the follower (Bass et al., 1987).

Second, in addition to role modeling charismatic behaviors, leader charisma has an affective component that is essential to the understanding of the charismatic relationship (Pescosolido, 2002). Indeed, charisma was initially conceptualized as “an emotional form of communal relationship” (Weber, 1947, p. 360). Charismatic individuals tend to feel and display positive affect (Erez et al., 2008), such as smiling, laughing, and being warm and affable (Cherulnik et al., 2001), and to express positive emotions when formulating their vision (Bono and Ilies, 2006).

The charismatic leader’s displays of positive affect are associated with positive affect among followers or with the latter’s affective tone (Bono and Ilies, 2006; Erez et al., 2008; Johnson, 2009; Sy et al., 2018). This emotional contagion is “a process in which a person or group influences the emotions or behaviors of another person or group through the conscious or unconscious induction of emotion states” (Schoenewolf, 1990, p. 50). As highly salient group members, charismatic leaders have major effects on their followers’ emotions (Johnson, 2009) and the emotional contagion is particularly strong (Connelly et al., 2013). Specifically, positive affect is transferred both through the content that the charismatic leader conveys and through non-verbal affect manifestations (Gardner and Avolio, 1998; Ashkanasy and Tse, 2000). We propose that the leader’s positive affect influences the follower’s positive affect (i.e., contagion), which in turn leads to attributions of charisma to followers (Johnson, 2009).

Third, the desired charismatic identity images stem from the leader’s self-concept (Gardner and Avolio, 1998). Self-concept is the multidimensional set of ideas about who one is, in terms of content, attitudes, or evaluative judgments, that one uses to make sense of the world (Oyserman, 2001). The self-concept is shaped by an individual’s unique experiences with their environment (Markus and Wurf, 1987). Thus, it is a flexible and dynamic construct that encompasses a variety of self-schemas tied to specific social situations and contexts (Lord et al., 1999; van Knippenberg et al., 2004). Charismatic leaders exert influence on their followers’ self-concept by framing and aligning meaning (Shamir et al., 1993), which links followers to a possible idealized collective self. In other words, a leader’s charisma may flow to followers by transforming the latter’s self-concept.

By means of positive evaluations, the placement of emphasis on an intrinsic aspect of rewards, demonstrations of confidence in the followers’ abilities, and communication of high-performance expectations, the charismatic leader can elevate their followers’ self-worth, self-esteem, self- and collective- efficacy, self-consistency, and collective identity (Shamir et al., 1993). The increased salience of collective identity and value internalization contributes to the followers’ commitment to the collective mission and fosters the communication of shared values, the use of “we” and “us” languages, and the willingness to make personal sacrifices for the group’s mission (Shamir et al., 1993). All these characteristics are attributed to charismatic leadership (Conger and Kanungo, 1987). Thus, we hypothesize the following:

Hypothesis 1: Charisma flows from leader to followers - the leader’s charisma positively contributes to the followers’ charisma.

### The flow of charisma: self-monitoring as a moderator

The extant literature on the trickle-down effect of leadership focuses mainly on the effect of certain extraneous factors, such as the organizational climate (e.g., Mawritz et al., 2012; Shin, 2012), on the leadership transmission process, while paying insufficient attention to the role of individual characteristics. Followers’ individual characteristics determine their differential response to role models or their susceptibility to the leader’s charisma (Liden and Antonakis, 2009; Wegge et al., 2022). One dispositional attribute associated with self-status enhancement and, therefore, with leadership emergence is self-monitoring (e.g., Day et al., 2002; Eby et al., 2003; Türetgen et al., 2008). Self-monitoring is the extent to which a person is able and willing to observe, regulate, and control their behaviors and public self-presentation in alignment with the situational requirements and expectations of others (Snyder, 1979; Kudret et al., 2019). Self-monitoring may serve as a boundary condition to the flow of charisma for two reasons: First, the Adapted Elaboration Likelihood Model (AELM; Wo et al., 2019) suggests that factors influencing individuals’ motivation and ability to process social information, such as self-monitoring, play a pivotal role in determining the flow of trickle effects. Specifically, self-monitoring, which reflects the inclination to attend to external cues, could function as a recipient-related characteristic that moderates the flow of charisma. Second, prior research has demonstrated the relevance of self-monitoring in relation to leadership emergence (e.g., Day et al., 2002; Sosik et al., 2002), and theorists and researchers have been encouraged to further consider how self-monitoring is associated with charisma (Kudret et al., 2019).

In a social situation, a high self-monitoring individual asks, “Who does this situation want me to be and how can I be that person?” (Snyder, 1979, p. 102). In this process, the high self-monitors assess the situational context to discern the ideal persona demanded by that specific situation. They then create a mental representation of a person who embodies that ideal character and employ this prototypical figure’s self-presentation and expressive actions as a reference framework for monitoring their own verbal and non-verbal behaviors. While a low self-monitoring individual, who is usually referred to as an “authentic self” (Bedeian and Day, 2004), would ask “Who am I and how can I be me in this situation?” (Snyder, 1979, p. 103). Much like the high self-monitors who assess the situation’s nature and subsequently form an illustrative persona for behavioral guidance, the low self-monitors follow a similar process. However, instead of crafting a representation of the prototypical individual suited for the context, the low self-monitors rely on a lasting self-image or self-perception that encapsulates their typical actions in the behavioral realms pertinent to the situation. This established self-image then functions as the operational framework guiding the low self-monitoring individual’s monitoring of their actions.

Since self-monitoring captures interpersonal variation in the degree to which individual behavior reflects interpersonal cues as opposed to inner affective states, self-monitoring has been treated as a moderator of the effects of other traits (Barrick et al., 2005), contributing to a finer-grained understanding of individual behaviors. However, research on high self-monitors has revealed inconsistent results regarding the nature of their social interactions. On the one hand, some studies demonstrated that high self-monitoring is associated with promoting positive social interactions and success in life and work (Tasselli et al., 2015; Kudret et al., 2019). As skilled impression managers (Snyder, 1979), high self-monitors tailor and fashion their emotions and behaviors to what they perceive to be the “correct image of a leader.” Since high self-monitors are affected by social cues (Kudret et al., 2019), they are prompted by the leader’s attitudes and behaviors and look at the leader as a role model for guiding their own attitudes and behaviors. This sensitivity to the leader’s cues may lead to like-acting behaviors to fit into the specific social context.

However, on the other hand, other researchers have called high self-monitoring individuals “chameleons” (e.g., Bedeian and Day, 2004; Scott et al., 2012) and noted that high self-monitoring may also be an essential component in eliciting negative social activities, such as lying, concealing one’s true intentions (Gangestad and Snyder, 2000), performing emotional manipulation (Grieve, 2011), displaying a low level of honesty-humility (Ogunfowora et al., 2013), and presenting an inauthentic self (Gangestad and Snyder, 2000). Furthermore, high self-monitors’ absorption of the charismatic leader’s displays of positive affect (i.e., the emotional contagion) may be a superficial imitation of the leader’s charismatic emotional cues, because high self-monitors tend to simulate their emotional expressions without actually feeling them (Brotheridge and Lee, 2002; Diefendorff et al., 2005). Moreover, the tendency of high self-monitors to alter their behaviors to fit the situational demands harms the consolidation of a stable identity (Gore and Cross, 2011), and thus harms their ability to communicate a consistent vision on key issues (Day et al., 2002). These, in turn, are associated with high self-monitors being perceived as inconsistent, self-promotional, and lacking personal integrity, and as not accurately and consistently reflecting their authentic selves (Bedeian and Day, 2004), thereby, decreasing the perception of the higher self-monitoring individual as authentically charismatic.

Low self-monitors are also sensitive to contextual cues, although, their motivation to use such cues in shaping their self-presentation is different (Shaffer et al., 1987). They look inward and rely on their own dispositions, beliefs, and attitudes to guide their behaviors, thus demonstrating consistency between their inner states and self-presentation (Day et al., 2002; Kudret et al., 2019). They may use the charismatic cues as an inner guide to self-transformation and as a prime to modify internal states if these cues are congruent with their own self (DeMarree et al., 2005). Furthermore, since low self-monitors are more likely to act in accordance with their inner self, and they may be more responsive to charismatic cues when these are perceived to convey information about their self-characteristics or emotions, the leader’s charismatic cues serve as diagnostic self-information that exerts large effects (Fiske and von Hendy, 1992). These effects alter their self-concept and, in that way, modify their emotions and behaviors (Lippa, 1978). Hence, low self-monitors behave in ways that accurately and consistently reflect their self-conceptions (Day et al., 2002) and are perceived as highly authentic, thereby, increasing the perception of the lower self-monitoring individual as authentically charismatic. We hypothesize the following:

Hypothesis 2: Follower’s self-monitoring moderates the flow of charisma from leader to followers.

## Materials and methods

### Sample and procedure

The field experiment was conducted in a large government security organization in Israel. A total of 151 trainees from across the organization had attended a 12 weeks training program, after which they returned to their original departments. The training program aims to develop several skills and abilities through a variety of learning methods such as lectures, simulations, and team projects that require trainees to take formal responsibilities for the completion and success of the tasks. In addition, note that the training program is intense in the sense that participants are required to spend 24/7 together with their group peers and group leader, thus providing ample opportunity for participants to become well-acquainted with one another.

The study protocol was approved by the authors’ institutional review board and received all the necessary permissions from the organization^2^. The research data was collected at two time points: on the second day of the training program (T1) and 5 weeks later, in the middle of the training program (T2). Upon arrival at the training program, the participants were randomly placed into 17 different teams and all were invited to participate in the study. Each team had been randomly assigned a different formal team leader who was an officer in the training program staff. The team leader’s role was to command the team, deliver part of the course content, lead and instruct the team in operational tasks, and accompany them in working on the team projects. This intensive course involved an average of 16 hours of leader-follower contact daily 127 participants from 15 teams took part in T1^3^. In each team, the number of participants ranged from 7 to 13 (*M* = 8 participants per team). Out of the 127 participants who responded to the questionnaire at T1, 82 responded to the follow-up questionnaire at T2 (i.e., 35.43% attrition rate). To assess potential differences between the participants at T1 and T2, we conducted a multivariate analysis of variance (MANOVA) with self-monitoring, extraversion, and average participant’s evaluation of teammates’ charisma at T1 as the dependent variables and participation in the study at T2 as an independent variable. There were no significant differences between the samples (*F*_(3_,_83)_ = 0.40, *ns*). Note that, although team leaders’ and participants’ charisma were collected at T2, overall, the final sample included 127 participants since the 82 participants at T2 evaluated the charisma of their team leader and all the other team members.

At T1 the participants received a link to an electronic survey that included the control variables, a self-monitoring scale, and a sociometric questionnaire in which they were asked to evaluate each of their team members on the charismatic leadership scale^4^. At T2, the participants received another link with the following questionnaires: the sociometric charismatic leadership scale (same as in T1) with which they evaluated each of their team members, and the same scale to evaluate their team leader’s charismatic leadership. Team leaders were not present in the room while participants completed their questionnaires privately. The match between the respondents’ answers to the surveys at T1 and T2 was made possible by allocating a unique identifying code to each respondent.

The participants’ ages ranged from 20 to 25 years (*M* = 21.58, *SD* = 1.13 years), and 55.6% were female. Tenure in the organization ranges from 9 to 64 months (*M* = 21.18, *SD* = 8.63 months). Most participants had a high school diploma (88.0%) and 12% had academic degrees. Team leaders’ ages ranged from 22 to 26 years (*M* = 23.4, *SD* = 1.06 years), and 66.67% were female.

### Measures

#### Charismatic leadership

The team leaders’ charismatic leadership was assessed at T2 using the Conger-Kanungo scale (C-K scale; Conger et al., 2000). The scale was translated into Hebrew, following the back-translation procedure. This scale includes the charismatic dimensions of strategic vision and articulation, sensitivity to the environment, sensitivity to members’ needs, personal risk, and unconventional behavior. Since the strategic vision and articulation items were not congruent with the specific training context, we did not include these dimensions in the questionnaire. Thus, the final scale included 13 items (e.g., “Our team leader takes high personal risks for the sake of the team”; “Our team leader uses non-traditional means to achieve team goals”), was used to assess the participants’ evaluations of their team leader at T2. Since we were interested in understanding the overall effect of charismatic leadership, we combined the four dimensions into one charismatic leadership scale (α = 0.95). We aggregated individual evaluations of a team leader to the team level, because leadership is not necessarily an individual perception and can be elevated to a shared perception as a team construct (Carter et al., 2013). In order to justify the aggregation of the individual perceptions to the team level, we calculated agreement indices. Results revealed a mean *R*_*WG*_ = 0.82, *ICC*(1) = 0.24 and *ICC*(2) = 0.65. Overall, these results warrant consideration of the leader charisma scale as a shared team construct.

The followers’ charismatic leadership was assessed at T1 and T2 using the same C-K scale with a referent to “My team member X [name of the team member]” (α = 0.97 and α = 0.95, respectively). Each participant was evaluated by their team members, and their charismatic leadership score was calculated as the average of these sociometric evaluations. In all scales, responses ranged on a 5-point Likert scale from 1 = “very uncharacteristic” to 5 = “very characteristic.”

#### Self-monitoring

Self-monitoring was measured at T1 using the Revised Self-Monitoring Scale (Lennox and Wolfe, 1984). We used the Hebrew version (Delegach and Katz-Navon, 2021). The scale included 13 items (e.g., “I have the ability to control the way I come across to people, depending on the impression I wish to give them,” α = 0.81) and ranged on a 5-point Likert scale from 1 = “certainly not, always false” to 5 = “certainly, always true.”

#### Control variables

We controlled for the leader’s experience as a team leader in the training program, which refers to the number of training programs they had led and ranged from 1 to 4 programs. This data was collected using the organizational database. Additionally, we controlled for followers’ extraversion, which refers to the tendency to be assertive and social, to experience positive affect, and to seek excitement (McCrae and John, 1992). This construct was previously found to be particularly related to charismatic leadership (e.g., Oreg and Berson, 2015). Extraversion was assessed using 8 items (α = 0.79) from the Hebrew version of the Big Five Inventory (John et al., 1991; Etzion and Laski, 1998) on a 5-point Likert scale.

## Results

Table 1 presents the means, standard deviations, and correlations among the study variables. First, we conducted a three-factor model CFA, including the items of extraversion, self-monitoring, and participant’s charisma measured at T1. The measurement model fitted the data well, with χ^2^(482) = 638.22, *p* < 0.001, CFI = 0.93, TLI = 0.92, and RMSEA = 0.05. Note that the results should be interpreted with caution since the observation-to-parameter ratio (*N/q*) was 3.7, whereas a ratio of 10 is usually recommended (Kline, 2016).

**TABLE 1:** Means, standard deviations, and correlations among the study variables.

	*M* (*SD*)	1	2	3	4	5
1. Leader experience as a team leader	0.42 (0.27)					
2. Follower extraversion	3.64 (0.57)	0.20*				
3. Follower self-monitoring	3.83 (0.45)	0.13	0.31**			
4. Leader charisma	4.04 (0.52)	0.34**	−0.08	0.10		
5. Follower charisma (T1)	3.95 (0.41)	0.17	0.09	−0.03	−0.02	
6. Follower charisma (T2)	3.88 (0.39)	0.35**	0.09	0.01	0.42**	0.17^+^

*N* = 127, ^+^*p* < 0.10, *p < 0.05, **p < 0.01.

Given that our data was collected at two points in time, wherein multiple measurements over time were nested within individuals and had a hierarchical structure in which individuals were nested within teams, we used a multilevel repeated measures approach using IBM SPSS (Version 23) Mixed-models procedure. Changes in follower charisma from T1 to T2 were represented as a within-person variable by the inclusion of time as a predictor, indicating the extent to which the charisma changed in a single person over time.

**FIGURE 2:**
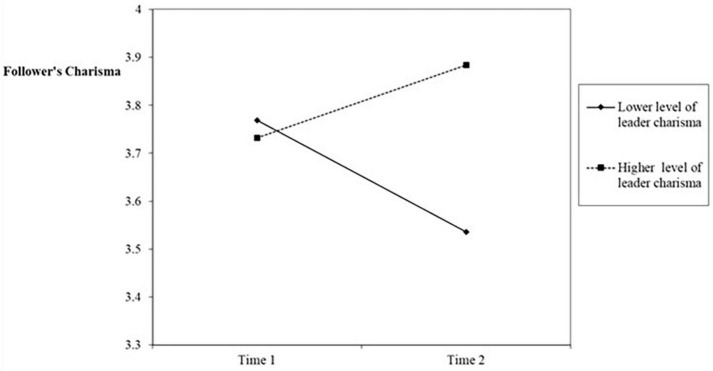
Followers’ charisma as a function of time.

In order to test Hypothesis 1, we conducted the following mixed-model regressions: First, we regressed the follower’s charisma on the control variables (leader’s experience and follower’s extraversion) and the two main effects (leader’s charisma and time, see Table 2, Model 1). Then we added the two-way interaction of leader’s charisma and time (Table 2, Model 2). Model 2 differed significantly from Model 1 (Δ-2loglikelihood = 18.4, *p* < 0.01). Results demonstrated a significant two-way interaction (*B* = 0.37, *SE* = 0.08, *p* < 0.001). Following the procedure illustrated by Aiken and West (1991), we plotted in Figure 2 the interaction at conditional values of leader charisma (1 SD above and below the mean). A simple slope analysis for multilevel models (Preacher et al., 2006) indicated that when the leader’s charisma was higher, it had a significant beneficial effect on their followers’ charisma (*B* = 0.23, *SE* = 0.15, *z* = 2.69, *p* < 0.01) and when the leader’s charisma was lower, it had a significant detrimental effect on their followers’ charisma (*B* = −0.23, *SE* = 0.05, *z* = −4.25, *p* < 0.01). These results support Hypothesis 1.

**TABLE 2:** Multilevel mixed models analyses of follower charisma on time, leader charisma, and follower self-monitoring.

Effect	Model 1 *B* (s.e.)	Model 2 *B* (s.e.)	Model 3 *B* (s.e.)	Model 4 *B* (s.e.)
Intercept	3.73** (0.17)	3.73** (0.17)	3.80** (0.26)	3.76** (0.26)
Leader experience as a team leader	0.20 (0.23)	0.20 (0.23)	0.20 (0.22)	0.19 (0.22)
Follower extraversion	0.03 (0.04)	0.03 (0.04)	−0.03 (0.06)	0.05 (0.04)
Follower self-monitoring			−0.03 (0.07)	−0.03 (0.06)
Leader charisma	0.15 (0.12)	0.14 (0.12)	0.17 (0.12)	0.46 (0.45)
Time	−0.05 (0.04)	−0.04 (0.04)	−0.06 (0.04)	0.32 (0.39)
Time × Follower self-monitoring				−0.10 (0.11)
Time × Leader charisma		0.36** (0.08)		1.96** (0.72)
Follower self-monitoring × Leader charisma				−0.08 (0.12)
Time × Follower self-monitoring × Leader charisma				−0.44* (0.20)
Random variance Team	0.04	0.04	0.04	0.04
Random variance Team × Subject	0.01	0.02	0.01	0.02
-2loglikelihood	187.95	169.49	177.18	154.79

*N* = 127, *p < 0.05, **p < 0.01.

**FIGURE 3:**
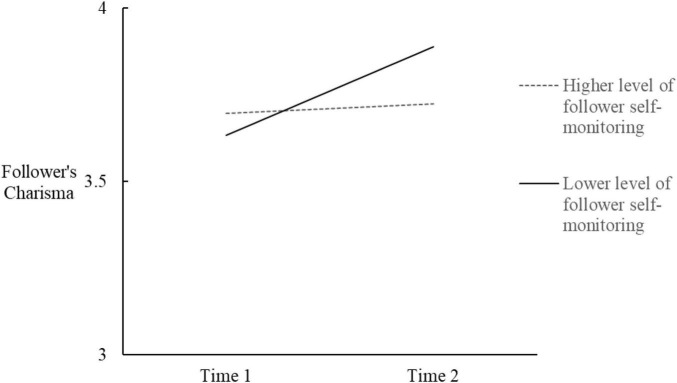
Followers’ charisma as a function of time and follower self-monitoring, when the leader’s charisma was higher.

**FIGURE 4:**
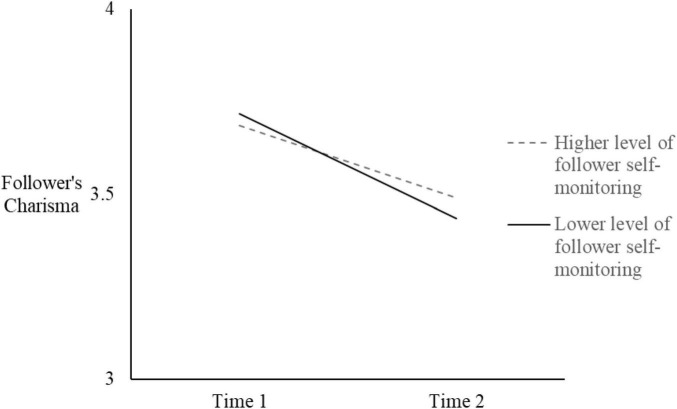
Followers’ charisma as a function of time and follower self-monitoring, when the leader’s charisma was lower.

Next, in order to test Hypothesis 2, we added the moderator (follower’s self-monitoring), the two two-way interactions, and the three-way interaction of leader’s charisma, time, and self-monitoring (see Table 2 Models 3 and 4). Results demonstrated a significant three-way interaction (*B* = −0.44, *SE* = 0.20, *p* = 0.03). Model 4 differed significantly from Model 2 (Δ-2loglikelihood = 14.70, *p* < 0.05) and from Model 3 (Δ-2loglikelihood = 22.39, *p* < 0.01). Moreover, according to Becker’s (2005) recommendation for the treatment of control variables, we reran the analysis without the latter. The three-way interaction term revealed the same pattern of results (*B* = −0.44, *SE* = 0.20, *p* = 0.03). The three-way interaction is plotted in Figures 3, 4. [Aiken and West (1991), at conditional values of leader charisma and self-monitoring as 1 SD above and below the mean]. A simple slope analysis for multilevel models (Preacher et al., 2006) indicated that when the leader’s charisma was lower, it had a significant detrimental effect on their followers’ charisma, both for higher (*B* = −0.29, *SE* = 0.08, *z* = −2.48, *p* < 0.05) and lower self-monitors (*B* = −0.28, *SE* = 0.07, *z* = −4.11, *p* < 0.01) followers. When the leader’s charisma was higher, its beneficial effect on followers’ charisma was apparent for lower self-monitoring followers (*B* = 0.26, *SE* = 0.07, *z* = 3.48, *p* < 0.01), but not for higher self-monitoring followers (*B* = 0.03, *SE* = 0.07, *z* = 0.40, *ns*).

## Discussion

Based on the positive association between charismatic leadership and followers’ performance (Banks et al., 2017), it is imperative for organizations to develop their leaders’ charisma. Several different approaches to charisma development are mentioned in the literature (e.g., Holladay and Coombs, 1993; Towler, 2003; Antonakis et al., 2011), focusing mostly on the active training of managers with specific charismatic behaviors and techniques. The current study suggests a more implicit process of charisma acquisition and emphasizes whether and when charismatic leadership flows down from the leader to the followers. The results of the current study revealed that charisma cascaded from the leader to their followers. Specifically, as hypothesized, throughout time, followers of a highly charismatic leader increased their charisma, compared to followers of a less charismatic leader. Furthermore, followers’ self-monitoring was found to be a boundary condition for the flow of charisma from leader to followers such that, when the leader’s charisma was higher, the cascading effect was prominent only for lower self-monitors. When the leader’s charisma was lower, a “negative” cascading effect took hold such that the charisma of both higher and lower self-monitoring followers decreased throughout time, though the lower self-monitors were still perceived as more charismatic than the higher self-monitors.

These findings contribute to the leadership, charisma, and self-monitoring literatures. First, there is ample literature on charisma as a trait-based individual quality (e.g., Riggio, 2009; Vergauwe et al., 2018). The current study results (Hypothesis 1) demonstrated the flow-down effect of charisma as a way to develop followers’ charisma. This sheds light on the socially learned process of charisma in which followers adopt their leader as a role model for specific charismatic behaviors, attitudes, and emotions. Second, most previous cascading leadership research assumes similarities between leaders’ and followers’ leadership styles due to an imitation process (e.g., Mayer et al., 2009; Mawritz et al., 2012; Wang et al., 2018; Xie et al., 2019). However, another potential explanation may be that the cascading effect occurs due to differential selection—followers are either self-selected or selected by their manager so that they are compatible with the manager’s leadership style (Bass et al., 1987; Schaubroeck et al., 2012). The attraction-selection-attrition theory (Schneider, 1987) supports the latter, suggesting that managers favor those who are similar to themselves when screening potential employees (Nielsen and Nielsen, 2011). The current study refutes this alternative explanation by the lack of pre-selection or consideration of leaders and followers and by tracking the relation between leaders and followers through time, from the moment they meet each other.

Third, adding to the literature on the trickle-down effect of positive leader behaviors (e.g., Bass et al., 1987; Mayer et al., 2009), we demonstrated that followers also emulate less charismatic leaders. These results expand the flow-down effect of non-charismatic leaders who happen to serve as role models for less constructive leadership styles. This suggests that leadership research may advance by expanding the focus from positive leader behaviors to combined leader behaviors of different valences, and studying how they may jointly or differentially trickle down through various organizational levels to affect followers’ attitudes, affect, and behaviors.

Finally, the study reveals additional refinements of the flow-down effect of charisma by identifying the moderating effect of the self-monitoring motive (Hypothesis 2). The study’s results enable us to more accurately understand the role of self-monitoring in the charisma flow-down process. As such, our research offers valuable insights into the boundary condition of the cascading contagion effects of charisma. Furthermore, it may seem counterintuitive that lower self-monitors, who habitually rely on self-knowledge, exhibit greater self-change in response to their leader’s charismatic signaling than higher self-monitors. However, charisma is in the eyes of the beholder (Conger and Kanungo, 1987); higher self-monitors may have changed too, but may be perceived as less genuine and thus as less charismatic. Future research may explore this proposition and shed light on the interplay between charisma development and the self-monitoring motive. In addition, it should explore a wider variety of follower motives and traits that might moderate the flow-down effect of charisma. Specifically, research should focus on additional follower traits that have important implications for the effectiveness of various leadership behaviors, for example, promotion focus (Delegach et al., 2017) or Machiavellianism (Deluga, 2001).

## Limitations and future research

Although the current study used a semi-experimental design in an organizational setting and a time lag sociometric measure for followers’ charisma, several of its limitations are noteworthy because they are most likely to open up venues for future research. One of the notable strengths of this study lies in its ability to investigate the phenomenon in its natural context. This approach ensures both external and ecological validity, as it allows us to observe and measure the phenomenon in a real-world environment. Moreover, it allowed for a certain level of causal inference. However, these strengths were counterbalanced by feasibility constraints: Logistical challenges related to the training program dictated the timing of our measurements. This could potentially introduce limitations, as the timing of measurements can influence the accuracy of detecting the effect. It might lead to underestimation, overestimation, or even the failure to detect certain effects (Timmons and Preacher, 2015). As a result, future research in this area could benefit from measuring the trickle-down effect at different time intervals and with longer trajectories. This approach would help investigate whether the developed charisma is a permanent characteristic or if it tends to decay over time or when the charismatic leader is no longer in close proximity to the followers. Such investigations would provide valuable insights into the temporal aspects of the trickle-down effect of charisma. Furthermore, the feasibility factor also imposed restrictions on the size and nature of the sample. A larger sample would likely yield substantial improvements in the overall reliability of the results, and to enhance the external validity of the study results replications in different organizational settings are warranted.

In addition, in the current study, we focused on demonstrating the existence of the flow-down effect of charisma, but we know little about what explains it. Future research should explore which mechanisms are involved in the cascading of charisma from leaders to followers. The flow-down effect of charisma represents a complicated dynamic social influence process; hence, future research may explore multiple mechanisms at work simultaneously. Moreover, as both the source and the recipient are critical parties in the flow-down effect of charisma, attention should be paid not only to the followers but also to the leader’s personal characteristics and to the contextual variables that affect the latter as well.

Furthermore, the current study focused on self-monitoring as a moderator of the trickle-down effect of charisma. Future research may consider other individual differences as moderators of the trickle-down effect of charisma. One such factor to consider is core self-evaluation (Johnson et al., 2008). The concept of core self-evaluation, as a personality trait, was initially introduced by Packer (1985), who defined it as “basic conclusions, bottom-line evaluations, that we all hold subconsciously. These evaluations pertain to three fundamental areas of every person’s life: self, reality, and other people” (p. 3). Unlike self-monitoring, core self-evaluation is a broader concept stable over time and across situations that encompasses an individual’s fundamental beliefs and evaluations about themselves. It comprises components, including self-esteem, self-efficacy, locus of control, and emotional stability (Judge et al., 1997). Thus, this construct reflects a person’s overall self-worth and self-perception, delving deeper into how a person views themselves concerning their abilities, self-worth, and control over their life. Hence, both constructs differ in their focus and implications: Self-monitoring pertains to adaptive behaviors and sensitivity to external cues, while core self-evaluation, encompasses a broader set of self-beliefs that reflects a person’s overall self-concept. Followers with higher core self-evaluation may exhibit behaviors consistent with their traits and effectively leverage the potential benefits of their high core self-evaluation for personal self-enhancement and the improvement of their charisma.

In addition, future research may consider contextual elements as moderators of the trickle-down effect of charisma. For example, Leader-Member Exchange (Graen and Uhl-Bien, 1995). Specifically, followers in high LMX relationships who interact more than followers in low LMX relationships with their leaders may increase opportunities to observe, attend to, and emulate their leaders’ charisma.

Finally, a great portion of the literature on the trickle-down effect of leadership refers to at least three organizational hierarchies, in which the source influences the recipient indirectly through a transmitter. The current study essentially focuses on the source and the transmitter only. Future research should explore the potentially longer chain of charisma flow through additional organizational levels and the differential effect of close and distant charismatic role models (Antonakis and Atwater, 2002). Finally, a replication of our results in a different organizational context would further strengthen the generalizability of our findings.

## Practical implications

The findings of this research offer practical implications for both managers and practitioners. To begin with, organizations can invest in leadership development programs with a specific focus on enhancing leaders’ charisma. This investment can have a positive cascading effect, gradually improving the charisma of their followers over time. Moreover, leaders can act either as transmitters or as inhibitors of charisma. Therefore, organizations should implement regular feedback mechanisms to gauge the perceived charisma levels of leaders and followers. This allows for the early detection of issues and enables timely interventions, including charisma training for less charismatic leaders, to prevent any adverse impact on their followers’ charisma.

Furthermore, organizations should acknowledge that not all followers respond uniformly to charismatic leadership. Therefore, it’s essential to customize charisma development strategies according to followers’ self-monitoring tendencies. For followers with high self-monitoring tendencies, providing additional leadership support and development can be advantageous. Alternatively, organizations can design training programs that not only enhance leadership charisma but also educate leaders on adapting their charisma to align with the self-monitoring tendencies of their followers. This tailored approach can lead to more effective leadership and better engagement of followers.

In sum, charismatic leaders can have a lasting positive impact on their followers and the organization as a whole. Thus, when selecting leaders or planning leadership development programs, organizations should consider the charisma factor.

## Data availability statement

The raw data supporting the conclusions of this article will be made available by the authors, without undue reservation.

## Ethics statement

The studies involving humans were approved by the Reichman University School of Psychology Review Board. The studies were conducted in accordance with the local legislation and institutional requirements. The participants provided their written informed consent to participate in this study.

## Author contributions

TK-N, MD, and EH designed the research. EH performed the data collection. TK-N and MD performed the data analyses and wrote the manuscript. All authors contributed to the article and approved the submitted version.

## Footnotes

1 Trickle-down effect research points to the indirect social influence of a source on a recipient through a transmitter (Wo et al., 2019). The current study focuses on the direct social influence of the leader on the transmitter (e.g., Johnson et al., 2017).

2 The data are available from the corresponding author upon reasonable request.

3 A power analysis to determine the sample size was not conducted before collecting data, since we relied on the maximum number of trainees in the training program available to the study at the time.

4 Signaling theory (Antonakis et al., 2011) suggests that people use cues transmitted by leaders to form impressions of charisma. Specifically, perceptions of charisma from thin slices of behavior (verbal and non-verbal) relate to inferences of charisma. Indeed, as research has shown, even brief observations of 5−30°s of non-verbal behavior can predict the perceived charisma of an individual (Tskhay et al., 2017).
